# Systemic Lupus Erythematosus with Hepatosplenic Granuloma: A Rare Case

**DOI:** 10.1155/2014/737453

**Published:** 2014-11-20

**Authors:** Anju Bharti, Lalit Prashant Meena

**Affiliations:** ^1^Department of Pathology, King George Medical University, Lucknow 226003, India; ^2^Department of General Medicine, Institute of Medical Sciences, Banaras Hindu University, Varanasi 221005, India

## Abstract

*Background*. Systemic lupus erythematosus (SLE) is an autoimmune disease which is known to present with a wide variety of clinical manifestations. *Case Report*. A 15-year-old male presented with complaints of moderate grade fever and generalized body swelling. There was no history of cough, weight loss, joint pain, oral ulcerations, skin rash, photosensitivity, loss of hair, pain abdomen, jaundice, or any significant illness in the past. Contrast enhanced computerized tomography of the abdomen revealed hypodense lesions in both liver and spleen (without contrast enhancement), suggestive of granulomas along with few retroperitoneal and mesenteric lymph nodes. On the basis of immunological tests and renal biopsy report, SLE with hepatosplenic granulomatosis diagnosis was made. He was given pulse methylprednisolone 500 mg, for 3 days and he showed dramatic improvement clinically. *Conclusion*. Hepatic and splenic granulomas are not common in SLE, but this should be kept in differential diagnosis.

## 1. Introduction

Systemic lupus erythematosus (SLE) is an autoimmune disease characterized by multisystem involvements. The exact etiology of systemic lupus erythematosus (SLE) is still not clear, although multifactorial interaction with environmental and genetic factors has been associated. Immune complex formation along with activation of complement system has been postulated for various manifestations of systemic lupus erythmatosus [1].

It is uncommon in children and young adolescents, with 0.5-0.6 per 1,00,000 per year being the incidence rate in children younger than 15 years of age. Moreover, it is rarer among male subjects [2]. Only very few reports have described noncaseating epithelioid cell granulomas in necropsy specimens of lymph node, lung, spleen, and serous membranes [3–5].

Although liver dysfunction may be found in SLE (25–50%), but SLE with hepatic and splenic granuloma is very rare. These features may also cause diagnostic confusion with other causes of granulomas. Here, we described a case presented with granulomas in liver and spleen.

## 2. Case Report

A 15-year-old male presented with complaints of moderate grade fever for 4 months and generalized body swelling with facial puffiness for 2 months. There was no history of cough, weight loss, joint pain, oral ulcerations, skin rash, photosensitivity, loss of hair, pain abdomen, jaundice, or any significant illness in the past. There was no past history of tuberculosis in the patient or in any other family member. General examination revealed moderate pallor and bilateral cervical and axillary lymph nodes, with the largest being of size 1 cm × 1 cm mobile, nontender, and firm. Systemic examination was insignificant except for the presence of palpable spleen, 2 cm below the costal margin. Hemogram was suggestive of bicytopenia (Hb 6.9 g/dL (normal: 12–14 g/dL), TLC 2700/mm^3^ (normal: 4000–11000/mm^3^), and total platelet count 2,10,000/mm^3^ (normal: 150000–400000/mm^3^)). Serum amino transferases and alkaline phosphatase were raised (SGOT 316 U/L (normal: 20–40 U/L), SGPT 109 U/L (normal: 20–50 U/L), and S. ALP 2750 IU/L (normal: 44 to 147 IU/L)). Total serum protein (5.1 g/dL, normal: 6.0–8.3 g/dL) and albumin (2.3 g/dL, normal: 3.5–5.5 g/dL) were low. Renal function tests were normal. Mantoux test was negative and ESR was slightly raised. Urine routine and microscopy showed 4-5 RBCs and 8–10 pus cells per high power field and 4+ albuminuria. 24-hour urinary protein was 2.1 gm. Kidney biopsy was done. Chest roentgenogram was normal. Ultrasonography of abdomen revealed mild splenomegaly with a tiny hypoechoic mass in spleen suggestive of splenic granuloma, along with a few subcentimetric retroperitoneal lymph nodes. Bone marrow studies were suggestive of nutritional anemia. Cervical lymph node biopsy showed reactive lymphoid hyperplasia. Barium meal follow through showed jejunoileitis. IgM antibody for Epstein-Barr virus was negative. Serum angiotensin converting enzyme levels were also normal. Based on the clinical picture, the high prevalence of tuberculosis in this part of the world and the investigations, a provisional diagnosis of disseminated tuberculosis was kept. The patient was started on a trial of antituberculous treatment and was asked for review with renal biopsy report. He turned up early with high grade fever, swelling of upper lips, and oral ulceration. On further investigations, his antinuclear and antidouble stranded DNA titers were significantly raised (ANA 7.3 IU/mL, N < 1, and anti-dsDNA 680 IU/mL, N < 40).

Histopathological report of kidney biopsy was suggestive of membranoproliferative (Type) lupus nephritis. Contrast enhanced computerized tomography of the abdomen revealed hypodense lesions in both liver and spleen (without contrast enhancement), suggestive of granulomas along with few retroperitoneal and mesenteric lymph nodes. Consequently, a revised diagnosis of SLE with lupus nephritis and granulomatous hepatitis was now evident, and the child was put on pulse methylprednisolone 500 mg for 3 days. He showed dramatic improvement clinically.

## 3. Discussion

**Figure 1:**
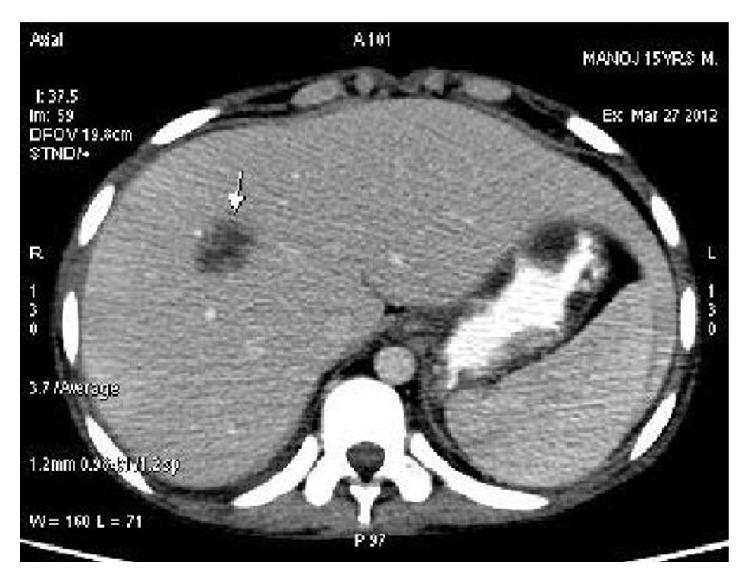
Hepatic and splenic granuloma.

**Figure 2:**
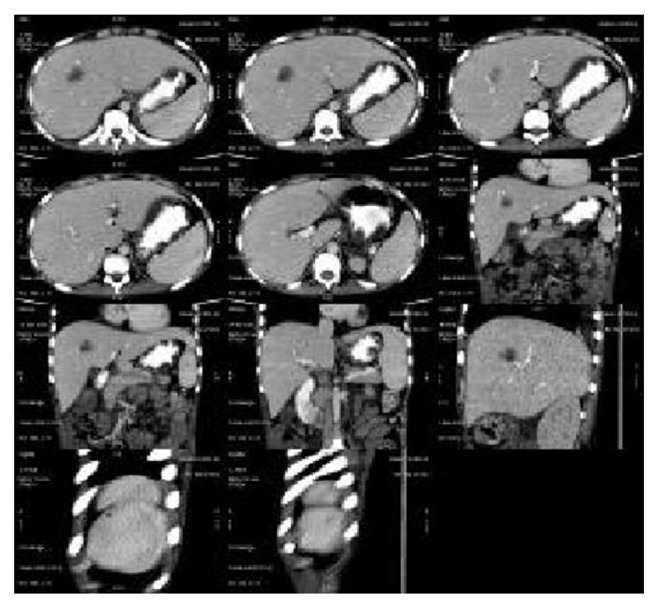
Hepatic and splenic granuloma.

SLE is an autoimmune disease that presents usually with multiorgan manifestations [2]. It is uncommon in children and young adolescents, with 0.5-0.6 per 1,00,000 per year being the incidence rate in children younger than 15 years of age. Moreover, it is rarer among male subjects. This coupled with the initial varied and vague presentation of the disease often leads to missing the diagnosis of the disease at first instance. In patients with SLE, subclinical manifestations or biochemical abnormalities of hepatic involvement are usually described, with overt disease seen rarely. Liver disease is diagnosed usually after one year of diagnosis of SLE. Various forms of hepatic involvement have already been described in SLE. Granulomatous hepatitis in SLE is uncommon, although not rare [6]. It is usually associated with raised hepatic enzymes, especially alkaline phosphatase levels and appears as hypoechoic/hypodense lesions on CT study. Starting the patient on corticosteroid therapy (as the treatment of SLE) is the main concern as the lesions closely mimic a liver abscess [7]. Presence of hepatic and splenic granulomas with generalized lymphadenopathy is well known in tuberculosis and sarcoidosis (Figures 1 and 2).

However, these were ruled out by relevant investigations. Possibility of an autoimmune disease like SLE was not kept initially despite the presence of significant proteinuria and active urinary sediments because of rarity of the disease in a male child, as described earlier. The only viable option was to place the patient on a trial of antituberculous treatment and look for response. However, failure to improve on ATT combined with the reports of renal biopsy, CECT abdomen, and autoimmune markers made the diagnosis of SLE imperative. Pathogenesis of granuloma formation in systemic lupus erythmatosus (SLE) is still not clear. It was postulated as a response to tissue injury and considered as a manifestation of allergic tissue reaction [3]. Although systemic lupus erythmatosus is considered type III hypersensitivity reaction, but type IV mediated mechanism also may play a role in autoimmune nephritis in the pathogenesis granuloma in SLE [8]. Reduced numbers of macrophages and capacity for phagocytosis along with defective clearance of apoptotic bodies by the complement system lead to a high level of apoptotic bodies in patients with SLE [9–11]. Hence, persistence of these apoptotic bodies may stimulate granuloma formation.

Therefore, in patients who present with granulomatous disease along with prolonged fever and constitutional symptoms, though uncommon, noninfectious causes like SLE should also be considered as a diagnostic possibility.
